# Relationships among serum C-reactive protein, receptor for advanced glycation products, metabolic dysfunction, and cognitive impairments

**DOI:** 10.1186/1471-2377-13-110

**Published:** 2013-08-27

**Authors:** Xia Ge, Xiao-yun Xu, Chun-hua Feng, Yue Wang, Yuan-ling Li, Bo Feng

**Affiliations:** 1 Department of Neurology, East Hospital, Tongji University, Shanghai, China; 2 Department of Neurology, Pudong New Area Zhoupu Hospital, Shanghai, China; 3 Department of Endocrinology, East Hospital, Tongji University, Shanghai, China

## Abstract

**Background:**

We examined the clinical value of two serum markers of low-grade inflammation, C-reactive protein (CRP) and receptor of advanced glycation products (RAGE), as prognostic indices for cognitive decline.

**Methods:**

Patients with cognitive impairment (n = 377) and controls (n = 66) were examined by blood biochemistry tests, including ELISAs of serum CRP and RAGE, the Mini-mental State Examination (MMSE) and Montreal Cognitive Assessment (MoCA), and STEAM 1H-MRS of the left hippocampus and thalamus.

**Results:**

Compared to the control group, the cognitive impairment group was older (63.10 ± 9.70 years vs. 55.09 ± 10.77 years, P = 0.000) and had fewer years of formal education (9.01 ± 4.01 vs. 12.94 ± 3.0, P = 0.000). There were no significant differences in the frequencies of type 2 diabetes, hypertension, or hyperlipidemia between groups. Serum CRP and RAGE were higher in the cognitive impairment group (CRP: 2.08 mg/L, range 1.07 − 3.36 mg/L vs. 0.21 mg/L, range 0.18 − 0.42 mg/L; RAGE: 4.01, range 2.49 − 5.71, vs. 2.28, range 1.84 − 3.03; P < 0.05 for both). In patients with cognitive impairment, there were negative correlations between cognitive function (as measured by MMSE and MoCA) and both CRP and RAGE levels (P < 0.05). Patients over 55 years exhibited a positive correlation between CRP and myo-inositol peak area in the left hippocampus (P < 0.05), while there was no relationship between RAGE and any metabolite (P > 0.05). Multiple linear regression revealed that CRP was influenced by hypertension (P = 0.026) and cognitive impairment (P = 0.042).

**Conclusions:**

Chronic low-grade inflammation is present in patients with cognitive impairment. Serum CRP, RAGE, and left hippocampal myo-inositol may provide prognostic information on cognitive decline.

## Background

Inflammation contributes to the development and exacerbation of cognitive decline in neurodegenerative diseases [1,2]. Individuals with C-reactive protein (CRP) concentrations > 3 mg/L are at significantly elevated risk for cardiovascular disease [3] while high-normal levels (2 ~ 3 mg/L) indicate low grade systemic inflammation [4]. Moreover, recent studies have established a correlation between serum CRP and cognitive impairment [5]. Another potential biological marker of cognitive decline, receptor for advanced glycation end products (RAGE), was first identified as a signal transduction receptor for the products of non-enzymatic glycation and oxidation of proteins and lipids (AGEs) that accumulate in diabetes and at inflammatory foci [6]. An association between RAGE levels and cognitive impairment in Alzheimer’s disease has been reported [7]. We evaluated the inter-relationships among serum CRP, serum RAGE, neuropsychological assessment scores, and cerebral metabolites associated with cognitive dysfunction to examine the role of chronic low grade inflammation in cognitive decline and metabolic changes in brain structures, such as the hippocampus, critical for cognitive function.

## Methods

### Patients

Patients assessed for cognitive decline at an out-patient department from September 2009 to November 2012 were recruited as the case group. Demographic parameters, including age and educational status, general medical condition as indicated by body mass index, personal and family histories of hypertension, type 2 diabetes, and stroke, and lifestyle risk factors such as smoking status, were recorded. Inclusion criteria included (1) cognitive decline for at least 6 months, (2) probable Alzheimer’s disease according to NINCDS-ADRDA criteria, (3) right handedness, and (4) Activity of Daily Living scale (ADL) score ≤ 26, Global Deterioration Scale (GDS) score ≤ 3, and Hamilton Depression scale (HAMD) score ≤ 7. Exclusion criteria included (1) sudden onset of cognitive decline, (2) history of neurological disease (e.g., large vessel stroke, Parkinson’s disease, severe brain injury, seizure, multiple sclerosis, brain infection, meningitis), (3) psychiatric illness (e.g., schizophrenia, bipolar disorder), (4) substance abuse (alcohol, drugs, or others), and (5) MRI contraindications. All the participants provided written informed consent before enrollment. In total, 377 patients were recruited as the patient group and 66 subjects without cognitive decline as the control group.

### Assessments

#### Blood biochemistry

Fasting blood concentrations of glucose, glycosylated hemoglobin, total triglycerides, total cholesterol, HDL-cholesterol, LDL-cholesterol, apolipoprotein A, apolipoprotein B, and apolipoprotein E were detected by standard enzymatic assays. Radial artery blood pressure was measured by a standard blood pressure monitor (VP-2000, Colin Medical Technology Corporation, Japan) after at least 10 minutes of rest. Standard height and weight measurements were used to calculated patient BMI.

#### C-Reactive Protein (CRP) and RAGE assays

Serum concentrations of CRP and RAGE were measured by ELISA using 4 mL fasting blood drawn from the cubital vein into coagulating tubes. Serum aliquots were stored at −80°C until analysis. Prior to analysis, the serum sample was diluted 50-fold. Commercial ELISA kits for CRP (Human High sensitivity) were obtained from USCN Life Science and RAGE kits from BlueGene.

#### Neuropsychological evaluation

Cognitive assessments included the Mini-mental State Examination (MMSE) and Montreal Cognitive Assessment (MoCA). The MMSE consists of 10 items testing orientation, memory, attention, calculation, language (naming, repeating, auditory comprehension, reading, and writing) and visual-spatial ability, for a maximum total score of 30. The MoCA tests 8 cognitive domains, visual-spatial ability, attention, executive function, immediate memory, delayed memory, language, abstraction, calculation, and orientation, for a maximum total score of 30. The normal MoCA score is ≥26, with one point added if the subject has fewer than 12 years of formal education. All tests were administered and scored by a professional trained in neuropsychometric testing.

#### Neuroimaging

1H-MRS parameters were as follows: echo time/repetition time TE = 9.4 ms, TR = 2000 ms, 128 excitations, TA = 4 min 48 s, volume 1.0 cm3 from the left hippocampal gray matter and 1.5 cm3 from the left thalamic gray matter. These regions were chosen because many metabolites measured in these areas are related to cognitive disorders. The chosen volumes were able to cover the ROI as more as possible without mix of cerebral spinal fluid or other region, and results of region smaller than those above would cause deviation for slight move from inevitable breath and heartbeat. The concentrations of N-Acetyl-L-aspartic acid (NAA), choline-containing compounds, myo-inositol, and glutamate were measured and reported as peak area. All images were acquired by a professional radiology technician.

### Data analyses

Data were analyzed using the Statistical Package for Social Sciences (SPSS 18.0). A two-tailed alpha level of 0.05 was the criterion for significance.

### Ethics

All the process was approved by the ethics committees of Shanghai East Hospital Affiliated to Tongji University and the study was operated in accordance with the World Medical Association’s Declaration of Helsinki.

## Results

A total of 443 participants were included in the final sample, 377 patients in the cognitive impairment group (178 men, 199 women) and 66 in the control group (38 men and 28 women). Demographic characteristics, indices of cardiovascular heath and metabolic disease, and serum CRP and RAGE concentrations are presented in Table 1.

**Table 1:** Demographics, cardiovascular and metabolic health status, and serum CRP and RAGE levels in cognitively impaired patients and controls

	**Cognitive impairment**	**Controls**	**Z**	**p**
	**(n = 377)**	**(n = 66)**		
**Age (year)**	63.10 ± 9.70	55.09 ± 10.77	−5.295	**0.000**
**Gender (male)**	178(47.2%)	38(57.6%)	−1.256	0.209
**Education (year)**	9.01 ± 4.01	12.94 ± 3.0	−6.455	**0.000**
**CRP (mg/L)**	2.08(1.07,3.36)	0.21(0.18,0.42)	−6.488	**0.001**
**RAGE (ng/mL)**	4.01(2.49,5.71)	2.28(1.84,3.03)	−3.271	**0.004**
**Hypertension**	60.5%	54.5%	−0.761	0.447
**Diabetes**	28.8%	38.7%	−1.348	0.178
**Triglycerides (mmol/L)**	1.38(0.89, 1.87)	1.49(0.99,2.06)	−0.831	0.406
**LDL (mmol/L)**	2.93(2.41,3.68)	3.22(2.67,4.18)	−1.651	0.099
**Smoking**	34.1%	34.8%	−0.151	0.880
**BMI (Kg/m**^**2**^**)**	23.58 ± 3.39	23.12 ± 2.63	−0.189	0.850

### Correlation among serum CRP, cognitive function, and cerebral metabolism

**Figure 1:**
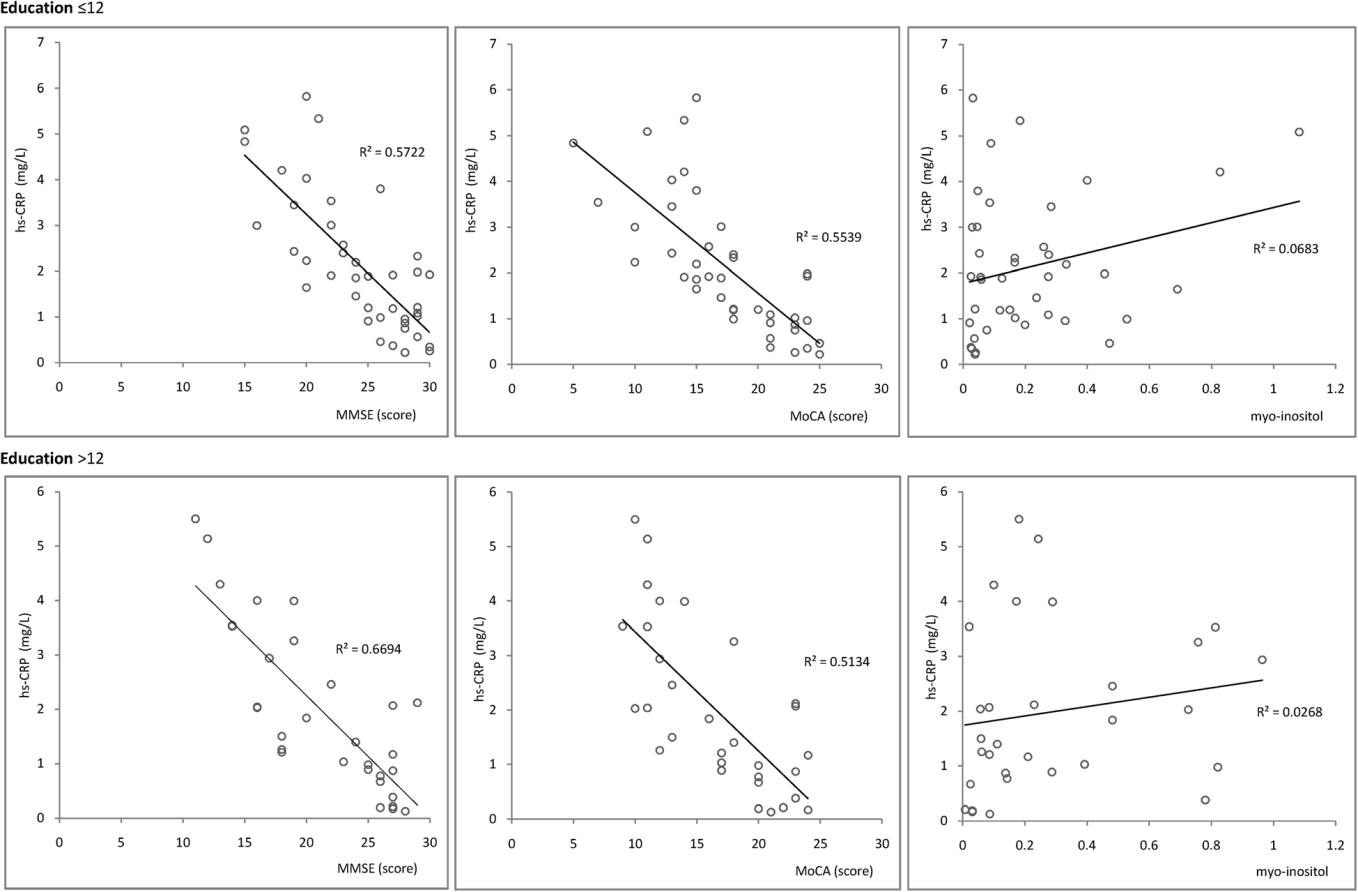
Correlations among serum CRP levels, cognitive assessment, and myo-inositol in Age range: 40–54.

**Figure 2:**
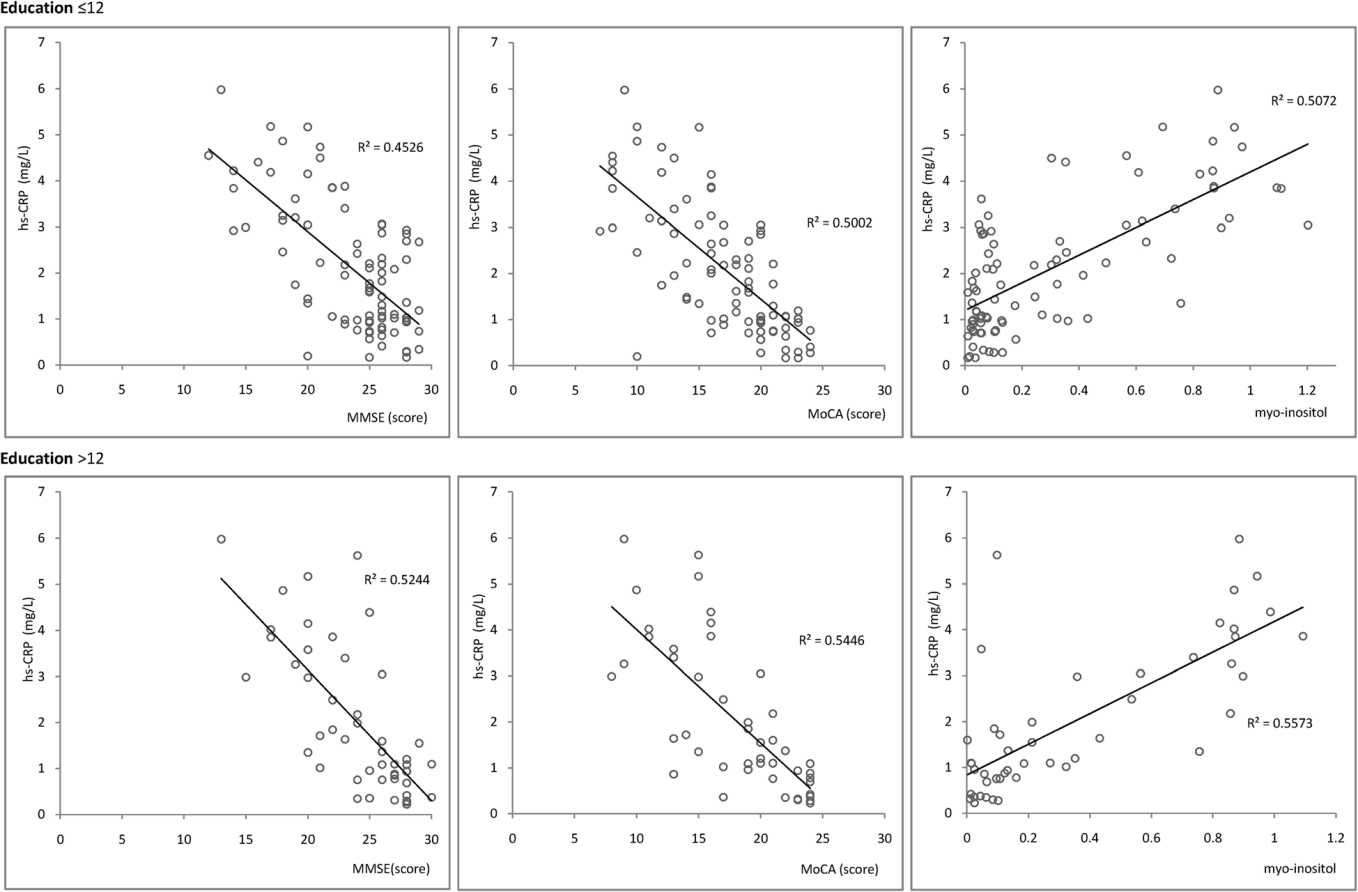
Correlations among serum CRP levels, cognitive assessment, and myo-inositol in Age range: 55–64.

**Figure 3:**
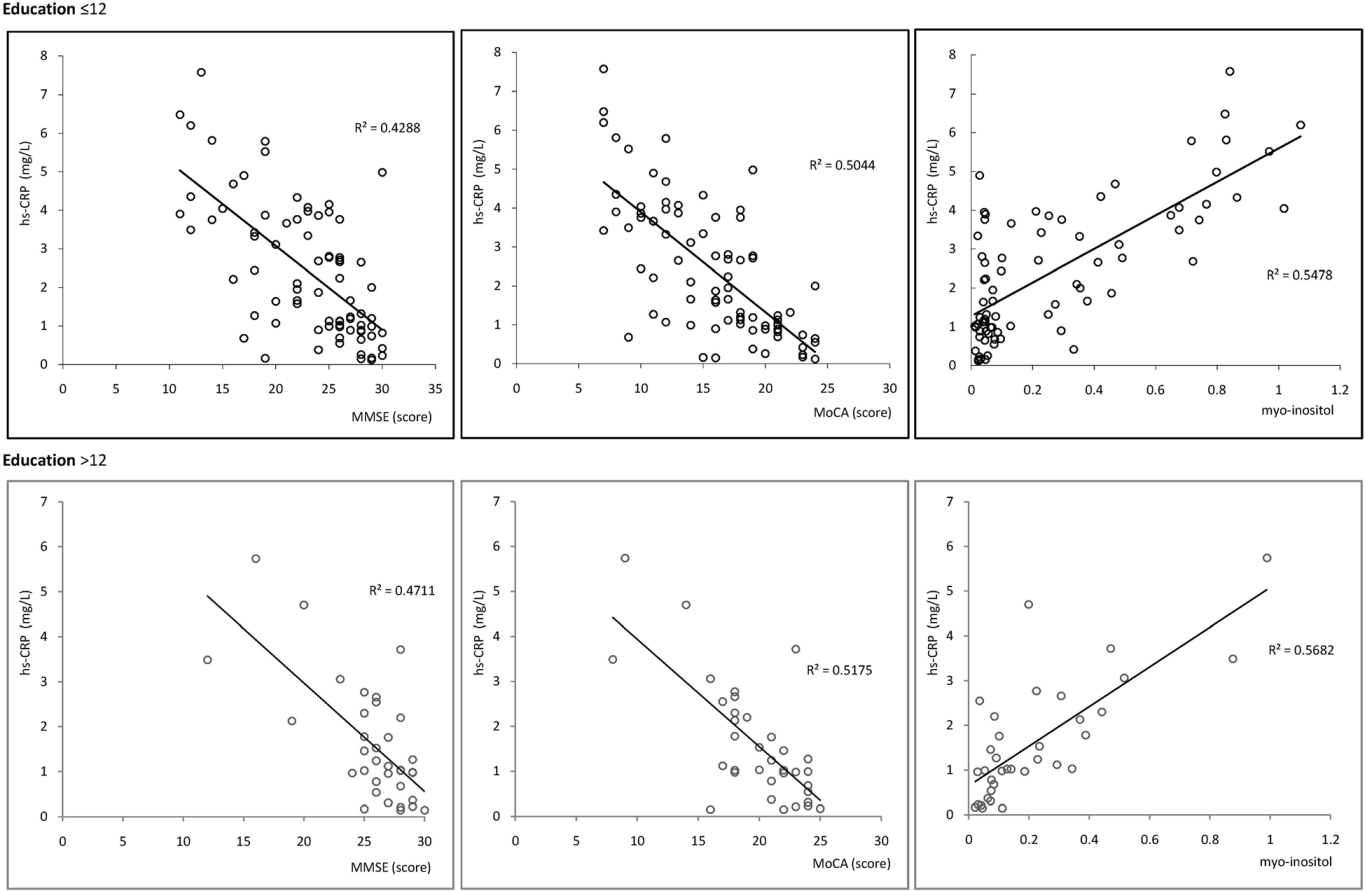
Correlations among serum CRP levels, cognitive assessment, and myo-inositol in Age range: 65–79.

**Figure 4:**
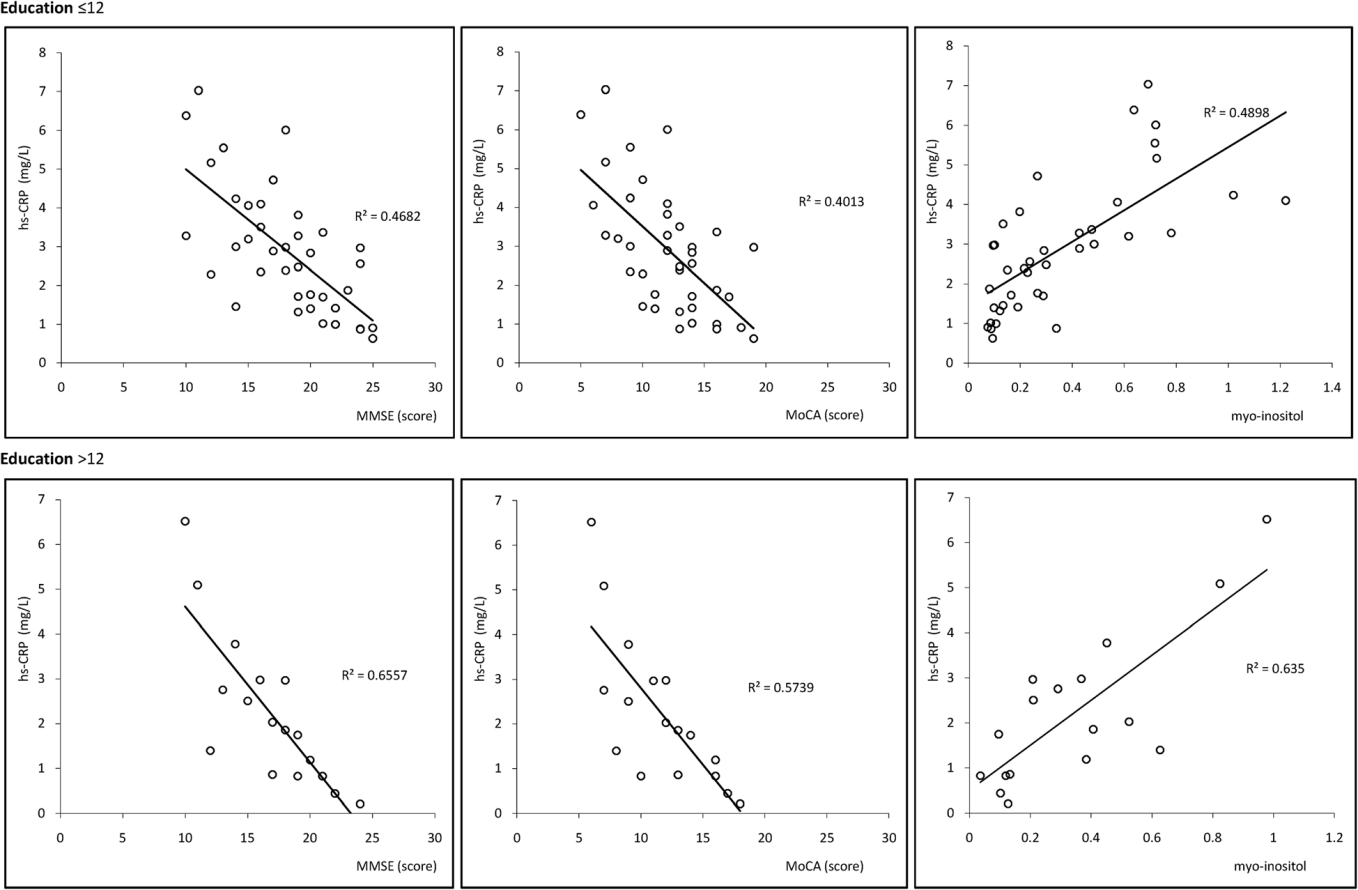
Correlations among serum CRP levels, cognitive assessment, and myo-inositol in Age range: >80.

Older age and lower education are generally accepted risk factors for cognitive dysfunction, and both were analyzed separately to avoid interference. Age was divided into 4 ranges: 40–54 (Figure 1), 55–64 (Figure 2), 65–79 (Figure 3), and > 80 years (Figure 4). Subjects were divided into two education groups according to whether they had 12 years of formal education, as this is the cut-off used to correct MoCA scores. Correlations among CRP, cognitive function, and cerebral metabolites were analyzed by Spearman correlation in the 8 subgroups defined by the four age groups and two education groups. Serum CRP was negatively correlated with MMSE and MoCA assessment scores (p < 0.05). There was a positive correlation between serum CRP and myo-inositol peak area in the left hippocampus (p < 0.05). Other metabolites measured by 1H-MRS were not significantly correlated with neuropsychological evaluation scores or CRP levels (Table 2).

**Table 2:** Correlations among serum CRP levels, cognitive function, and cerebral metabolism

**Age group**	**Education**	**n**	**To MMSE**		**To MoCA**		**To myo-inositol**	
**(years)**	**(years)**							
			**r**	**p**	**r**	**p**	**r**	**p**
**40 − 54**	≤12	41	−0.756	**0.025**	−0.744	**0.015**	0.261	0.055
	>12	31	−0.818	**0.039**	−0.716	**0.019**	0.163	0.063
**55 − 64**	≤12	88	−0.672	**0.021**	−0.707	**0.011**	0.712	**0.026**
	>12	47	−0.724	**0.022**	−0.738	**0.023**	0.747	**0.029**
**65 − 79**	≤12	79	−0.655	**0.017**	−0.710	**0.015**	0.740	**0.018**
	>12	35	−0.686	**0.021**	−0.719	**0.027**	0.754	**0.021**
**>80**	≤12	39	−0.684	**0.034**	−0.633	**0.028**	0.670	**0.036**
	>12	17	−0.810	**0.036**	−0.758	**0.031**	0.797	**0.041**

(Figures 1, 2, 3 and 4).

### Correlations among serum RAGE, cognitive function, and cerebral metabolism

Serum RAGE was negatively correlated with MMSE and MoCA scores (p < 0.05). However, there was no significant negative correlation between serum RAGE and NAA peak area as predicted, or with any other metabolic changes in the left hippocampus (Table 3) or thalamus (p > 0.05) as measured by 1H-MRS.

**Table 3:** Correlations among serum RAGE, cognition function, and cerebral metabolism

**Age group**	**Education**	**n**	**To MMSE**		**To MoCA**		**To NAA**	
**(years)**	**(years)**		**r**	**p**	**r**	**p**	**r**	**P**
**40 − 54**	≤12	41	−0.511	**0.032**	−0.625	**0.024**	−0.212	0.063
	>12	31	−0.598	**0.047**	−0.456	**0.037**	−0.194	0.058
**55 − 64**	≤12	88	−0.563	**0.041**	−0.604	**0.028**	−0.235	0.072
	>12	47	−0.492	**0.038**	−0.462	**0.033**	−0.186	0.062
**65 − 79**	≤12	79	−0.637	**0.029**	−0.693	**0.019**	−0.242	0.103
	>12	35	−0.552	**0.032**	−0.721	**0.025**	−0.255	0.096
**>80**	≤12	39	−0.648	**0.030**	−0.723	**0.028**	−0.267	0.078
	>12	17	−0.562	**0.026**	−0.820	**0.034**	−0.321	0.089

### Multiple liner regression analysis of factor related to CRP

The effects of serum CRP and RAGE on cardiovascular parameters did not interact, so each was analyzed separately by multiple liner regression. There were no significant associations for RAGE, while stepwise regression showed that CRP was affected by hypertension (p = 0.026) and cognitive impairment (p = 0.042). The coefficient of determination of the regression analysis indicated that all the variables (Table 4) accounted for 60.3% of the variability in serum CRP (p = 0.028).

**Table 4:** Multiple linear regression analysis of CRP

**Independents**	**B**	**Std. Error**	**Std. Coefficients(β)**	**t**	**p**
**b0**	5.456	1.163	-	2.691	0.020
**Gender**	0.085	0.326	0.087	0.578	0.556
**Age**	0.045	0.201	0.399	0.305	0.762
**Education**	−0.052	0.213	0.434	−0.319	0.751
**Hypertension**	0.469	0.224	0.324	2.096	**0.026**
**Diabetes**	1.343	0.788	0.565	1.251	0.064
**Triglyceride**	−0.003	0.121	0.382	−0.016	0.987
**LDL**	0.158	0.354	0.255	1.075	0.289
**Cognitive**	2.245	0.969	0.294	2.316	**0.042**
**Smoking**	0.210	0.263	0.339	1.457	0.153
**BMI index**	0.096	0.342	0.278	0.647	0.521

R^2^ = 0.0603, F = 8.266, P = 0.028.

## Discussion

The clinical diagnosis of early stage cognitive impairment depends primarily on neuropsychological assessments because there are often no obvious structural lesions or metabolic changes in the brain as revealed by neuroimaging. Prognostic markers predictive of cognitive decline in the earliest stages prior to measureable impairments may facilitate timely intervention. Low grade inflammation is a possible contributing factor to cognitive decline and as such, indices of inflammation may be indicative of early pathogenic processes in brain regions associated with cognitive function. Pathological changes in the brain are often not reflected by changes in peripheral blood chemistry due to the blood brain barrier (BBB), but neural inflammation may be manifested by changes in peripheral blood if the BBB is disrupted by the pathology. Thus, it is possible that inflammatory mediators in peripheral blood may be the earliest signs of neurodegenerative disease. With this information as background, our target was to investigate the relationship between peripheral systemic inflammation and cerebral metabolism in AD cognitive decline.

Indeed, we found that inflammation as reflected by serum CRP was associated with cognition dysfunction. The median serum CRP in our cognitive impairment group was 2.08 mg/L, much higher than in the control sample (0.21 mg/L). Furthermore, CRP was inversely correlated with MMSE and MoCA scores in the total patient population and in subgroups stratified by age range and years of formal education (including the youngest higher-education group). Serum CRP is regarded as an independent risk factor for cardiovascular disease, and previous studies also suggested a relationship to cognitive decline [8-10], although no definitive mechanism has been established. If CRP is elevated before measurable cognitive decline, inflammation may contribute to cognitive impairment or accelerate the deterioration. For example, CRP can activate the complement system, causing a series of immune cascade reactions leading to neurodegeneration [11].

Serum CRP was also positively correlated with cerebral myo-inositol as indicated by 1H-MRS in subjects over 55 and independent of years of formal education. Myo-inositol is mainly found in astrocytes, and increases are indicative of glia proliferation as occurs in reactive gliosis [12]. Elevations of myo-inositol levels in Alzheimer’s disease patients have been interpreted as a sign of gliosis [13]. Astrocyte are phagocytic in the brain and spine and are induced to proliferate when the BBB is compromised. Potential mechanism of these results was supposed to associate with the “leakage” of inflammation. Low grade systemic inflammation mediated by CRP may lead to increased BBB permeability, impairment of endothelial function, and toxic elevations of intracellular calcium [14]. The intracellular second messenger inositol triphosphate(IP3) is not efficiently derived from myo-inositol and so may become depleted. This in turn would hinder calcium release and cause the failure of conduction through gap junctions between astrocytes, suppressing interstitial buffering functions and leading to neuronal hyperexcitability [15]. On the other hand, IP3 was found to associate with glioma by mediating signal transduction for activation and proliferation of gliocyte [16,17]. A compromised BBB may lead to release of inflammatory factors into the peripheral blood. Thus, the peripheral blood may contain markers indicative of neurodegenerative diseases like AD. But the precise connection between CRP and mI would be further studied and presented in future research.

Like CRP, RAGE is thought to impair cognition through an immune inflammatory pathway. Yan [18] first found 2.5-fold RAGE overexpession in AD brains. Evidence suggests that RAGE expression is enhanced in the hippocampus and frontal lobe, and that RAGE binds to β-amyloid (Aβ) [19]. β-Amyloid can permeate the BBB from circulating blood by combining with RAGE in endothelial cells [20], triggering amyloid aggregation and stimulating a complex chronic immune inflammatory response. This immune activation is neurotoxic and results in the pathological changes associated with AD [21]. The receptors RAGE, scavenger receptor, and serine protease inhibitor complex receptor (SEC-R) mediate the adherence of Aβ to microglia, which stimulates microglia activation. In turn, activated microglia release NO, TNF-α, TGF-β, and other cytotoxic factors [22]. Studies above all held the opinion that RAGE plays an important role in AD progression via immune and inflammation, while Different from the cellular RAGE measured by immunohistochemical measurement in tissue, what our study chose was a quantitative determination of its total level in serum, which stood for the measurable RAGE in peripheral blood. More efficacious RAGE existed in serum meant more potential immune and inflammation by RAGE antigen-antibody reaction would happen. Another popular sample sRAGE( soluble receptor for advanced glycation end products), the competitive receptor against RAGE, is supposed to be a protective factor on AD cognition [23]. We would further study it and other else relevant receptors or ligands in sequence.

Unexpectedly, we found no significant relationships between RAGE and diabetes, plasma glucose, or glycosylated hemoglobin. Cell activation due to AGE-RAGE interaction can occur in normal tissue via chronic immune inflammation, and this is aggravated in diabetes complication [24]. To date, however, the mechanism of cell activation in systemic diseases has not been defined. As expected, RAGE was much higher in the cognitive impairment group. Diabetes patients assigned in our study were the people without relevant complication, which might be involved in this result.

RAGE was not associated with any metabolic change in left hippocampus or thalamus. We predicted that RAGE may correlate with NAA, a sensitive neuroimaging biomarker indicative of neuronal integrity and function. In light of these results, we suggest that RAGE impairs cognition via immune signaling pathways that disrupt neuronal functions but do not damage neuronal integrity. Elevations in NAA are indicative of cellular microtrauma. In AD or cerebral ischemia concomitant with myocardial infarction, metabolic changes such as NAA reduction and myo-inositol elevation can be detected in the hippocampus [25,26]. We hypothesized that participants with cognitive impairment would have higher serum CRP/RAGE and lower NAA, but found no significant association between serum RAGE and any biomarker of cerebral metabolism. Whether RAGE can effect cerebral metabolism is currently unknown, and we found no evidence for such an effect. Nonetheless, RAGE was associated with cognitive impairment, so a RAGE-mediated immune inflammatory reaction may cause cognitive impairment by highly localized effects on neurocircuits.

Regression analysis revealed that hypertension and cognitive impairment led to the elevation of CRP in serum. Serum CRP is widely believed to be an independent predictor of heart attack and stroke as well as an independent risk factor for hypertension [27,28]. Results from animal experiments indicate that low grade inflammation can damage endothelial cells and reduce the generation of NO, which results in the overexpression of angiotensin receptor-1 (AR-1). Overexpression of AR-1 activates the renin-angiotensin-aldosterone system and causes hypertension [29]. However, the precise relationship between CRP and hypertension remains unclear. In our study cohort, most of the hypertensive patients were clinically obese with characteristics of metabolic syndrome. Hypertension, obesity, hyperlipidemia, and diabetes, the main manifestations of metabolic syndrome, do not always present simultaneously, while in obese patients, the presence of at least three of these manifestations can lead to CRP elevation [30]. Hypertension is a significant predictor of cardiovascular events and is strongly association with inflammation by CRP.

We found no interrelationship between the level of serum CRP and myo-inositol or other metabolites in the left hippocampus of hypertensives stratified by disease severity with or without cognitive impairment. Though CRP was higher in hypertension patients, there was no correlation between CRP (or RAGE) and brain metabolites related to inflammation. However, there has been little research on the influence of long-term chronic hypertension on the blood brain barrier and glial responses, so it is possible that CRP does not cross the BBB even if brain inflammation is present.

The cognitive impairment group was older than the control group and had fewer years of formal education, suggesting that a “cognitive reserve” in unafflicted individuals may prevent or mask cognitive impairment [31,32]. There were no significant differences in the frequencies of type 2 diabetes, hypertension, or hyperlipidemia between groups. Among these diseases, type 2 diabetes has been shown to negatively affect cognitive function [33] but there was no difference in diabetes incidence, plasma glucose, or glycosylated hemoglobin between groups. As metabolic syndrome progresses, diabetes coexists with hypertension, hyperlipidemia, and even obesity, leading to a low-grade inflammatory condition that ultimately impairs cognition [1]. The participants assigned to the cognitive impairment group were mostly in the early stages and demonstrated relatively mild impairments. Moreover, one of our objectives was to assess risk factors for cerebrovascular and cardiovascular diseases, and almost all patients accepted appropriate treatments. This early intervention may have contributed to the relatively mild effects on cognition.

## Conclusions

We found significant correlations between cognitive impairment and inflammatory markers in the peripheral circulation. Thus, we have identified a possible non-invasive method for detecting neuro-inflammation leading to cognitive dysfunction. One limitation of this study is that we only detected two markers. Future studies will assess additional markers, including interleukins, TNF, interferon, and complement together in a larger patient sample.

In sum, our data provide evidence that relatively high levels of serum CRP and RAGE have a negative impact on cognitive function. Additionally, there is a positive correlation between serum CRP and myo-inositol in the left hippocampus. Thus, serum CRP, RAGE, and myo-inositol in the left hippocampus are candidate biomarkers for cognitive diseases that warrant validation in future studies.

## Competing interests

The authors declare that they have no competing interest.

## Authors’ contributions

GX and XXY conceived the study and drafted the manuscript. GX also carried out all data analyses and wrote the paper. GX, FCH, WY and LYL participated in information collection, cognitive assessment, MRS scans and acquisition of data. XXY is the principal investigator for the overall program grant. All authors read and approved the final manuscript.

## Pre-publication history

The pre-publication history for this paper can be accessed here:

http://www.biomedcentral.com/1471-2377/13/110/prepub
